# The barriers and facilitators to the reporting and recording of self-harm in young people aged 18 and under: a systematic review

**DOI:** 10.1186/s12889-023-15046-7

**Published:** 2023-01-24

**Authors:** Gillian Waller, Dorothy Newbury-Birch, Diane Simpson, Emma Armstrong, Becky James, Lucy Chapman, Farhin Ahmed, Jennifer Ferguson

**Affiliations:** 1 NHS Business Services Authority, Stella House, Newburn, Newcastle, NE15 8NY UK; 2 School of Social Sciences, Humanities and Law, Teesside University, Middlesbrough, TS1 3BA UK; 3 Faculty of Education and Society, University of Sunderland, Sunderland, SR6 0DD UK; 4 Department of Health and Social Care, Office for Health Improvement & Disparities, Newburn, Newcastle, NE15 8NY UK; 5 Durham County Council, County Hall, Durham, DH1 5UJ UK

**Keywords:** Self-harm, Young people, Mental health, Recording, Reporting, Systematic review

## Abstract

**Background and aims:**

This systematic review sought to identify, explain and interpret the prominent or recurring themes relating to the barriers and facilitators of reporting and recording of self-harm in young people across different settings, such as the healthcare setting, schools and the criminal justice setting.

**Methods:**

A search strategy was developed to ensure all relevant literature around the reporting and recording of self-harm in young people was obtained. Literature searches were conducted in six databases and a grey literature search of policy documents and relevant material was also conducted. Due to the range of available literature, both quantitative and qualitative methodologies were considered for inclusion.

**Results:**

Following the completion of the literature searches and sifting, nineteen papers were eligible for inclusion.

Facilitators to reporting self-harm across the different settings were found to be recognising self-harm behaviours, using passive screening, training and experience, positive communication, and safe, private information sharing. Barriers to reporting self-harm included confidentiality concerns, negative perceptions of young people, communication difficulties, stigma, staff lacking knowledge around self-harm, and a lack of time, money and resources.

Facilitators to recording self-harm across the different settings included being open to discussing what is recorded, services working together and co-ordinated help. Barriers to recording self-harm were mainly around stigma, the information being recorded and the ability of staff being able to do so, and their length of professional experience.

**Conclusion:**

Following the review of the current evidence, it was apparent that there was still progress to be made to improve the reporting and recording of self-harm in young people, across the different settings. Future work should concentrate on better understanding the facilitators, whilst aiming to ameliorate the barriers.

## Background

Self-harm can be defined as an individual causing injury or poisoning to themselves, regardless of its intent [1]. It can include a plethora of different behaviours including hitting, cutting, poisoning or burning [1]. The presence of self-harm can be triggered by complex, heterogeneous factors, but it is commonly associated with mental illness, with individuals at an increased risk of suicide and attempted suicide. Prevalence rates of self-harm illuminate several at-risk groups when filtered by gender, region, ethnicity, and/or age. Public Health England’s (PHE) data shows that in 2019/20, 694.8 per 100,000 population of females and 196.6 per 100,000 population of males, aged 10–24 years, were admitted to hospital as a consequence of self-harm [2]. This disparity in self-harm rates between females and males remains consistent within prevalence estimates [3, 4], with McManus et al. [5] noting the greatest increase in self-harm rates being attributable to young women and girls. Similarly, the PHE data exposes considerable regional variations in hospital admissions resulting from self-harm in children and young people [2]. Such disparities have frequently been correlated to socioeconomic deprivation [6, 7], or discrepancies between the management of self-harm between hospitals [8, 9]. When comparing rates between ethnic groups, research indicates that black females are the most at-risk group [10, 11], though the data is generally limited in this area.

Young people and children are thought to be the most at-risk group, with rates generally declining with age after 25 years [5, 12]. Research has demonstrated that self-harm amongst children and adolescents in the UK has increased over the last two decades [13, 14], particularly for girls [15]. There have been several hypotheses as to why this increase has occurred. For example, one study has found increased rates of self-harm amongst adolescents with a friend who had self-harmed previously [16]. Additionally, Geulayov et al. (2022) [17] illuminated the link between loneliness during the Covid-19 pandemic and self-harm and point to the need for support schools and students as the repercussions of the pandemic continue. Yet, a systematic review found a reduction in service use by children and young people over the course of the pandemic [18]. It has been suggested that to prevent self-harm in children and young people, attention ought to be turned to issues that directly affect them such as bullying, mental health, family problems, and social media [19, 20]. However, as Borschmann and Kinner (2019) highlight, there is a lack of evidence documenting how effective interventions for this demographic would be [21].

Although determining prevalence is important in understanding and managing self-harm, it must be acknowledged that gaining accurate rates is inherently complex. To assess the difficulty of ascertaining prevalence rates, it is pertinent to consider the various streams of reporting and recording self-harm. First, some utilise statistics based on help-seeking via the GP. GP’s typically have a heavy workload and appointments are severely time limited [22]. Consequently, GP’s state that the screening tools for self-harm are too formal for a 10 min consultation, not allowing time for trust to be built between doctor and patient [23]. Secondly, hospital admissions have been used to discern self-harm rates. However, Hawton et al. [4] found that only 12.6% of the incidences of self-harm, reported by the 15–16 year old’s within their sample, required a visit to the hospital. Thirdly, one may rely on self-reported data, though Mars et al. [24] uncovered discrepancies within self-reported adolescent self-harm data, suggesting that prevalence approximations may underestimate the true rate. Lastly, research indicates that most children and young people who do seek help rely on informal streams of support [25, 26], in which case the self-harm may not be recorded at all. Evidently, gaining accurate prevalence rates requires extracting data from multiple sources.

Moreover, the existent literature demonstrates a variety of reasons why children and young people may choose not to report their self-harm, formally or informally. Those most in need of support for their suicidal ideation were the least likely to seek support [27]. For some young people, the belief that no external source can help or that the problem will resolve itself prevents them from seeking support [28, 29]. Other reasons included: not knowing who to confide in [26]; concerns about being placated with medication [30]; apprehensions around who to trust in terms of confidentiality, especially in rural areas [22]; and waiting times for seeing a health professional [22]. Biddle et al. [31] found that young men were less likely to seek support than young women, furthermore, the All-Party Parliamentary Group on Suicide and Self-Harm Prevention (APPGSS) [32] note that many struggle to access support, especially particular groups of young people such as those who identify as LGBTQ + , or those from an ethnic minority. Perhaps the most examined barrier to help-seeking, is the concern of stigmatisation.

Research has indicated that young people may not seek support out of fear of being labelled an ‘attention seeker’, by both peers and professionals [26, 33]. Utilising online tools, particularly anonymously, may enable those who are at-risk and not proactive in help-seeking to engage with some form of support system [34]. However, it has been suggested that the internet can normalise self-harm, with unrestricted access to gruesome imagery and new potential methods of harm [35]. The APPGSSP note that some worry they will be perceived as time wasters by health professionals as their injuries were self-inflicted, for some, this was based on their previous experiences of maltreatment [32]. Thus, the report recommends appropriate training for frontline staff, and a ‘buddy’ system within the NHS. Parker [36] found that the stigmatization of self-harming adolescents was perpetuated throughout schools through: word avoidance; topic avoidance; and negative judgmental behaviours. To combat these concerns around stigmatisation, the Royal College of Psychiatrists (RCP) discouraged health professionals from recording self-harm in a judgemental manner and to ensure all professionals have the right training and supervision [37]. Bailey et al. [38] found that the type of- and reason for- self-harm is often absent from patient records. To facilitate a move towards accurate recordings of self-harm in adolescents, Bailey et al. [38] recommend that health care professionals discuss with patients what is being written on their medical records. Overall, compounded with the issues above around barriers to reporting, our ability to access and analyse accurate prevalence rates is immensely restricted as a consequence of this guidance not to record reports of self-harm. The RCP [37] noted the need for self-harm training for staff in schools, however, it has since been established that the limited funding and resources available to schools bounds the scope for the implementation of such training [39].

This systematic review was proposed to further explore the barriers and facilitators to reporting and recording self-harm in young people and to identify gaps that still need addressing in future research and practice.

## Aim and objectives

The aim of this systematic review was to identify, explain and interpret the prominent or recurring themes relating to the barriers and facilitators of reporting and recording of self-harm in young people, across different settings, such as the healthcare setting, schools and the criminal justice setting.

This review sought to fulfil the following key primary objectives:

1. To identify, explain and interpret the prominent or recurring themes relating to the barriers and facilitators of reporting and recording of self-harm in young people,
2. To identify any gaps in the subject field in relation to young people reporting and recording self-harm,
3. To use the findings to inform qualitative work with both young people who have self-harmed and relevant practitioners.

## Methods

Due to anticipating the variable available evidence; the review was proposed as being best placed as a mixed-methods review. The SPIDER (Sample, Phenomenon of Interest, Design, Evaluation, Research Type) [40] tool was used to encompass both quantitative and qualitative searches and to ensure thorough searches were carried out. Table 1 presents an example of the SPIDER search terms that were used. Developing specific inclusion and exclusion criteria, relevant to the review's aims and objectives, assisted in selecting papers.

**Table 1:** Table of Search Terms by SPIDER [40]

Sample	Phenomenon of Interest	Design	Evaluation	Research Type (See design type)
Young people Adolescent* Teen* Teenager* Youth* Kids Aged 18 and under Not adults Girl* Boy* Minor* Young wom*n Young m*n Under 18	Self-harm* Self-harm Self-injury SH Self-violence Cutting Injury wound Self-inflicted violence Self-injurious behaviour Self-injurious behaviour Non-suicidal self-injury Physical harm Reporting Report* Describe Describing Story Detail* Statement Inform Information Account Recording Record Log Logging	Focus group* Interview* Observation* Ethnography Ethnographical Qualitative Survey questionnaire	Barrier* Challenges Walls Block* Obstacle* Obstruct* Hurdle* Difficulty Problem* Stop* Limit* Hinder* Facilitator* Facilitate Motivat* Enabl* Aid* Assist* Support* Allow* Permit* Ease* Further* Promote*	Mixed methods Qualitative Quantitative

* denotes truncation of a search term, which is used to ensure all variations of a search term are searched for

### Eligibility criteria

#### Inclusion criteria

Papers were eligible for inclusion in the systematic review if they presented barriers and facilitators to the reporting and the recording self-harm in young people. Self-harm, for the purpose of this review, was defined as "an act of self-injury or self-poisoning regardless of motivation or intent [41]”. The term reporting has been used to include the traditional concepts of help-seeking as any ‘any action or activity carried out by an adolescent who perceives herself/himself as needing personal, psychological, affective assistance or health or social services [42], whilst also including young people reporting their self-harm without the intention of receiving help to manage it. Recording has been used to include any method of documenting a young person’s self-harm, whether that it is in their medical notes, school files or within other social settings.

Papers were eligible if the population of interest was determined to be young people (males or females) aged 18 years and under. If any of the papers included a range of age groups (e.g., 12–20 years old), then they would only be eligible if the results relating to those aged 18 and under could be isolated. Any setting in which self-harm can be reported and recorded was acceptable for inclusion in the review. Therefore, it was anticipated that the review would include a range of settings and practitioners including schools, GP surgeries, hospitals, criminal justice settings etc. Both quantitative and qualitative and published and non-published literature were eligible for inclusion in the review, where relevant.

#### Exclusion criteria

Papers were not excluded by study design, location or language and any non-English papers were translated to assess relevance. Papers were excluded if self-harm could not be isolated from other behaviours and if young people were aged over 18 years. Studies published prior to 2004 were excluded as 2004 was the year self-harm was embedded into NICE guidelines and hence it is likely self-harm would have been recorded or reported differently within the literature [41].

### Search strategy

The search strategy was broad in order to capture all types of barriers and facilitators to the reporting or recording of self-harm in young people. Keywords were coupled with relevant MeSH/ thesaurus terms and truncated as appropriate. The following databases were searched: MedLine, PsycINFO, EMBASE, CINAHL, SCOPUS and the Cochrane Library. Studies published in any language, from any country were included from 2004 to the current day.

Grey literature was also searched. Searches were conducted on online platforms such as Google, Google Scholar, MedNar, opengrey.au and databases of conference proceedings. For Google, the first 10 pages of results were looked at to ensure the specificity of results returned and to avoid sifting through irrelevant material. In addition, relevant specialist websites were searched for potentially relevant literature including: Gov.uk, NSPCC, Barnardo’s, Royal College of Psychiatrists.

### Study selection

**Fig. 1:**
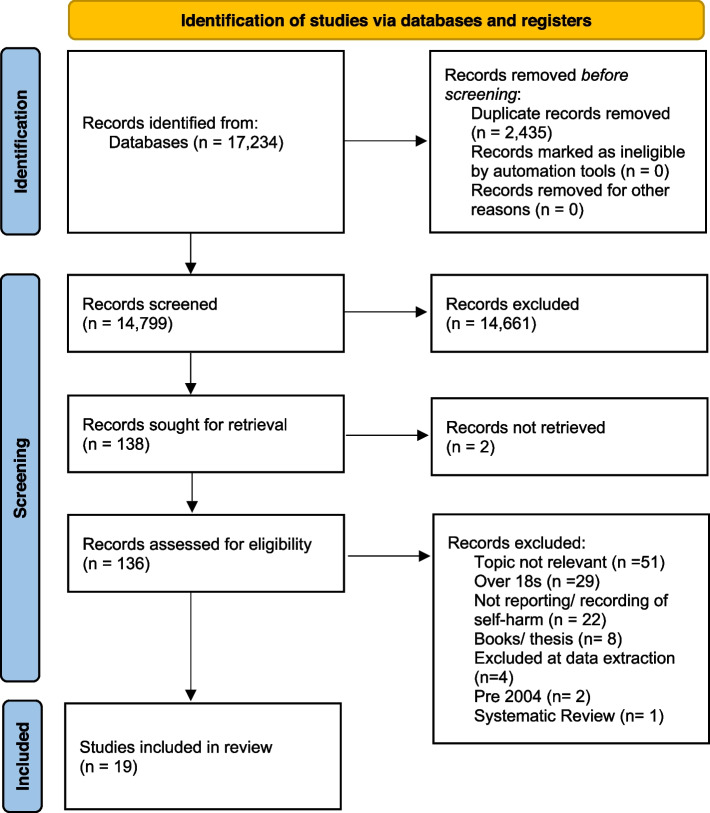
PRISMA Diagram depicting the flow of information through the different phases of the review

The Preferred Reporting Items for Systematic Reviews and Meta Analysis (PRISMA) flowchart (see Fig. 1) [43] was used as a guide and hence this review will be reported in accordance with the PRISMA statement [43, 44]. All search results, following the completion of the literature searches, across all six databases, were exported and managed in a newly created EndNote library. The first step was to remove any duplicate papers. Next, all of the titles and abstracts were screened against the review’s inclusion criteria by JF. For consistency, a second reviewer (DNB) double checked 20% of the search results, in order to determine whether decisions in relation to inclusion or exclusion matched. Any disagreements were recorded and resolved through team discussion. For the studies, that met the inclusion criteria, following the completion of the title and abstract screening, full text copies of the articles were retrieved for further exploration. These were then read and taken through to data extraction, if still appeared relevant. All full text articles were checked by two reviewers independently (GW and JF), and any disagreements were resolved by bringing in a third reviewer (DNB).

### Data extraction and management

An Excel data extraction sheet was developed to extract relevant information from the included papers. To retain the focus of the paper and to avoid extracting irrelevant results, the extraction sheet was based around the following headings: Study, Country, Aim of Study, Type of Study, Participants, Setting, Facilitators to Reporting/ Recording and Barriers to Reporting/ Recording. For consistency, one researcher completed the data extraction (GW) and then a second team member (PA) went over the extraction sheet, to ensure no important findings had been missed or overlooked.

### Risk of bias (quality) assessment

To assess the quality of the included studies, the relevant Critical Appraisal Skills Programme (CASP) quality assessment checklists were used [45]. The CASP checklists determine whether the results of a paper are valid, what are the results and whether the results help locally by asking a series of questions around the risk of bias [45]. The purpose of using the CASP criteria was to assess paper quality, and hence it was not used to contribute to decisions about whether to include studies. Two reviewers independently applied the CASP criteria to the included studies (GW and PA), and any disagreements were recorded and resolved through discussion. High risk of bias was recorded if ‘no’ or ‘unsure’ were recorded for 6 or more of the 11 questions on the tool. Medium risk of bias was assigned if ‘no’ or ‘unsure’ were recorded for 4–5 questions and Low risk for 1–3 questions.

### Data synthesis

As it was anticipated that there would be a plethora of different study designs, the proposed synthesis was a narrative synthesis, which employed a thematic analysis. A narrative synthesis was used to ensure that all study types could be included in the review. The thematic synthesis was conducted to establish recurring and unique content across the studies that could be arranged into themes across the reporting and recording of self-harm. The thematic synthesis involved two reviewers coding the extracted data, identifying key themes, and categorising the themes that were established within facilitators or barriers of reporting or recording [46]. The key themes were then written up and presented as a narrative synthesis which all reviewers contributed to.

## Results

The literature searches were undertaken in November 2021. The initial database searches revealed 17,234 papers, with searches of the grey literature sources not yielding any additional results. After completing the first sift; 138 papers were taken into the full text screening phase. All the full text papers were obtained, and then following the second sift, 19 papers were deemed eligible for inclusion in the final review. Figure 1 shows a PRISMA Diagram which depicts the flow of information through the different phases of the review.

Nineteen papers were deemed eligible for inclusion. Ten papers were quantitative studies, using surveys or Delphi methodology [25, 47–55]. Seven papers were qualitative studies employing interviews or focus group methods [56–62]. One paper used mixed methods [63] and one paper was an editorial [23]. All papers were from high-income countries, with 8 studies being conducted within the UK, 3 within the US, 3 in Australia, 2 in Ireland, 1 in Finland, 1 in Norway and 1 in Canada. The included papers were based in four main settings: healthcare settings, schools, a criminal justice setting and online settings. Therefore, the extraction tables (Tables 2, 3, 4 and 5) were grouped by setting, to allow key factors and themes from each setting and the different providers to be realised. Table 2 consists of the seven papers focusing on exploring the factors affecting the reporting or recording self-harm within a healthcare setting, such as a hospital or a GP [23, 47–49, 56, 57, 62, 63]. Table 3 includes the nine papers based in a school setting [25, 50–54, 58–60]. Table 4 has one paper which was based in a criminal justice setting; a youth offending team [61]. The final table, Table 5, presents one paper which was focused around young people accessing online support and therefore encompassed a range of different providers, populations etc. [55]. The results of the quality assessment have been included in each of these tables.

**Table 2:** Table of the factors affecting the reporting or recording of self-harm within a healthcare setting

Study	Country	Aim	Type of Study	Participants	Setting	Facilitators to Reporting/Recording	Barriers to Reporting/Recording	Quality Assessment
Bailey et al. 2017 [23]	England	N/A	Editorial	N/A	GP	REPORTING (i) Giving the YP the opportunity to talk about their SH in their own words (ii) Guided self-help information- Worked through with GPs rather than being given to the YP to read after a consultation RECORDING (i) Talking to YP about what is recorded and for staff to be trained to support consistency of recording	REPORTING (i) YP having poor intrapersonal and communication skills (ii) YP having concerns about whether shared information will stay confidential and/or negative consequences of disclosure (iii) YP struggle to explain SH and fear not being taken seriously or respectfully because of age (iv) Short appointments not comprehensive enough. (v) Ineffective screening tools- Screening tools too formal for time-limited consultations, yet need to assess risks, whilst not exacerbating current risk or cause the YP more distress RECORDING (i) Stigma of DSH due its connotations of blame and associated stigma	MEDIUM RISK
Bellairs-Walsh et al. 2020 [57]	Australia	To explore YP's views and experiences related to the identification, assessment and care of suicidal behaviour and SH in primary care settings with GPs	Qualitative- Focus groups	Two focus groups with 10 YP	GP	REPORTING (i) Important to have a collaborative and ongoing dialogue (ii) YP want to be informed about sharing information to enhance feelings of comfort and safety. (iii) Language should be positive, inviting and warm. (iv) GPs should listen to YPs concerns, preferences and support them as an individual. (v) GPs displaying attentive body language, including eye contact and posture, and active listening. (vi) Rather than onus being on the YP, YP wanted GPs to initiate conversations	REPORTING (i) YP often had poor mental health literacy or felt hopeless or ‘like a burden’. (ii) YP described how failure by GPs to ask, could lead to missed opportunities. (iii) YP described fearing the consequences of disclosure, due to confidentiality and privacy of medical records and what may happen to information. (iv) YP viewed the language around risk as problematic, ‘negative’ and ‘intimidating'. (vi) An indifferent or impersonal attitude was seen as a barrier to honesty and disclosure. When risk-related assessments were a ‘tick-box’, formulaic manner, which could impact on the dynamics of the practitioner and patient relationship. This also hindered disclosure RECORDING (i) YP were worried about around what personal information was recorded	LOW RISK
Fisher & Foster, 2016 [63]	England	To develop an evidence-based care plan/ pathway for children and YP in paediatric inpatient settings presenting with SH/ suicidal behaviour	Qualitative and Quantitative- Delphi survey	5 junior and senior staff nurses	General Paediatric ward	REPORTING (i) Staff knowledge and understanding (ii) Being able to recognise behaviour when presented	REPORTING (i) Staff expressed need for further training due to suicidal behaviour being unpredictable (ii) Fear of making YP's difficulties worse. (iii) Negative perceptions of YP who SH- reported as disruptive, demanding, aggressive, and difficult to understand and communicate with. (iv) Difficult environment for disclosure- setting was busy, and chaotic with large workloads	LOW RISK
Hawton et al., 2009 [47]	UK	To compare the characteristics of YP who reported deliberate SH episodes and presented at a hospital with those not attending hospital	Quantitative Question-naire	6020 YP- 3186 males, 2810 females, 24 gender unknown)	Hospital	REPORTING (i) Previous Support- YP were more likely to present to hospital with DSH if they had sought help from parents, friends, or a psychologist/psychiatrist	REPORTING (i) Perceived severity of SH- Hospital presentation was rare following self-cutting, but more common after self-poisoning, other single methods of deliberate SH and multiple methods	LOW RISK
Jennings & Evans, 2020 [56]	Wales	To explore the YP SH management and prevention practices, following reports that multi-agency teams were not effectively operating	Qualitative- Interviews and Focus Groups	Residential carers (n = 15) and foster carers (n = 15)	Foster carers talking about their experience of clinicians in Wales	REPORTING (i) Clinicians learnt from experience not knowledge (ii) Distinguishing RC role from clinicians- exchange knowledge to further own knowledge on the YP	REPORTING (i) Not an individual approach- Clinicians were reliant on abstracted knowledge and did not always have a direct encounter with a YP. (ii) Not understanding YP- Often situations were complex with wider factors. (iii) Clinicians not respecting carers- Clinicians as dominant and not seeing other views/experience (iv) Negative perception of YP in care	LOW RISK
Miettinen et al. 2021 [62]	Finland	To describe experiences of help related to SH in YP	Qualitative Essays and Interviews	27 YP aged between 12–22 who had harmed themselves during adolescence	Different social contexts, and with different backgrounds in relation to treatment in Finland	REPORTING (i) *Access to range of professionals* to talk to and have continuity of care once in the system	REPORTING- (i) Access to Help- Getting an appointment was slow. (ii) Threshold to seek help- YP required many GP visits and multiple referrals (iii) YP were uncertain about the severity of their SH (being taken seriously) and requesting help. (iv) YP were unwilling to burden loved ones and unwilling to get help (v) Consequences- YP were afraid of disclosing and not being taken seriously. (iv) Parents were often reluctant about a YP’s need for treatment RECORDING- (i) Avoidance- YP reported injuries were ignored, despite being asked. (ii) Not Recording- Professionals were unable to deal with SH and recording. Staff were reported as not reacting after having seen SH	LOW RISK
Saini et al., 2021 [48]	England	To use Delphi methodology to reach consensus between different stakeholders and researchers on research priorities in suicide and SH to develop regional SH and suicide prevention and reduction schemes	Delphi Method	88 conference attendees-clinicians, researchers, experts, police, third sector workers, commissioners and pharmacists	The Suicide and Self-harm Research North West (SSHARE NOW) conference	REPORTING (i) Training for those working with YP in non-medical settings, such as schools or community settings. (ii) Help-seeking- Understanding more about how and when YP seek help REPORTING and RECORDING (i) Services working together- The communication between different services and how they can work together can facilitate processes	REPORTING (i) Stigma. (ii) Lack of knowledge from organisational practices	MEDIUM RISK
Tørmoen et al., 2014 [49]	Norway	To explore child and adolescent psychiatric services (CAPS) with both suicide attempts and non-suicidal SH, and to assess the psychosocial variables of YP	Quantitative Question-naire	11,440 YP aged 14–17 years	75 of 91 junior and senior high schools	REPORTING (i) Service Use- YP who reported SH were more likely to have used CAPS	REPORTING (i) A YP with a non-Western immigrant background was associated with a lower likelihood of accessing CAPS. (ii) In YP with both suicide attempts and NSSH, symptoms of depression, eating problems, and the use of illicit drugs were associated with a higher likelihood of CAPS contact	LOW RISK

**Table 3:** Table of the factors affecting the reporting or recording of self-harm within a school setting

Study	Country	Aim	Type of Study	Participants	Setting	Facilitators to Reporting/Recording	Barriers to Reporting/Recording	Quality Assessment
Berger et al., 2014 [50]	Australia	To validate a measure of attitudes towards NSSI and examine the knowledge, attitudes, and confidence of staff towards NSSI	Quantitative- Questionnaire	501 secondary school staff- 261 teachers; 106 MH workers, counsellors, psychologists and welfare coordinators; 82 school leaders and 52 admin and support staff	86 Secondary schools	REPORTING (i) Helpful Staff- Willingness to help YP by resolving education needs and helping them manage emotions RECORDING (i) Younger staff were more knowledgeable and had higher self-perceived knowledge of NSSI than older colleagues	REPORTING (i) Lack of knowledge and confidence to assess and refer. (ii) Education- Need more around helping YP to seek help RECORDING (i) Length of professional experience-senior staff had poorer knowledge around identifying NSSI (ii) Females reported greater confidence and NSSI knowledge (iii) Experienced staff were more confident and had higher knowledge, understanding and more positive attitudes	LOW RISK
Dowling & Doyle, 2017 [58]	Ireland	To explore post-primary school guidance counsellors’ and teachers’ experiences of and responses to self-harm among students	Qualitative- Interviews	6 participants (all female) from 4 schools. 3 were guidance counsellors and 3 were teachers including a year head and a principal	All school types (i.e., all-boys, all girls and mixed) in areas of both high and low social deprivation	REPORTING- (i) Being able to Identify SH- Noticing subtle behaviour changes or being told about SH by YP or friends/ family. (ii) Different ways of Reporting- Disclosure to English teachers happened through essays and PE teachers noticed a YP’s refusal to change or wearing bandages to hide injuries. (iii) Not knowing a YP well made it easier for staff to cope with. (iv) Staff Debrief-To manage their own emotions staff drew upon colleague support, family and other self-care strategies	REPORTING- (i) Hard to identify- YP used ‘jumpers’, ‘uniforms’ or ‘bandages’ to cover up. (ii) Staff felt panicked by SH reporting, but this reduced with experience. (iii) Hard to Deal With- SH described as ‘difficult’, ‘horrible’, ‘disturbing’. (iv) Confidentiality (v) Negative Perceptions- Staff were less tolerant for advantaged YP and perceived them as ‘attention seeking’ (vi) Larger class sizes and fewer, busier teachers resulted in less time for SH reporting (vii) Inexperienced staff struggled, felt out of their depth, overwhelmed and anxious	LOW RISK
Evans et al., 2005 [25]	UK	To explore whether YP who deliberately harmed themselves or had thoughts of SH differed from others in terms of help-seeking, and coping strategies	Quantitative- Questionnaire	6020 15–16 year old school pupils, were surveyed using an anonymous self-report questionnaire	41 schools (35 comprehensive, 4 independent and 2 grammar) from 3 areas in England	REPORTING (i) YP were most likely able to talk to a friend (84.7%), followed by mothers (67.0%) and YP with a single episode of SH were more able to talk to relatives and friends than YP with multiple DSH episodes (ii) Females were more likely to reach out for help	REPORTING (i) YP did not feel comfortable to their teachers with only 20.8% reporting they would be able to talk to them. (ii) Ability to identify SH as an issue- Quarter of the YP did not feel they had a problem with SH	LOW RISK
Evans et al., 2019 [51]	UK	To understand school context-existing provision, barriers to implementation, & acceptability of different approaches	Quantitative- Questionnaire	222 schools in England and Wales were invited to participate 68.9% (n = 153) responded	Secondary schools		REPORTING (i) Lack of training was only identified as being moderate (ii) Limited Time/ Resources	MEDIUM RISK
Heath et al., 2010 [52]	Canada	To examine YPs reports of willingness to access school based NSSI support	Quantitative- Questionnaire	7,126 middle and high school students	Schools from 11 school districts in Greater Kansas City metro area	REPORTING (i) No significant difference between females and males		LOW RISK
Nearchou et al., 2018 [53]	Ireland	To determine predictors of YP help-seeking intentions for symptoms of depression/ anxiety and SH	Quantitative- Questionnaire	722 Participants (n = 368 girls)	3 cohorts of secondary schools		REPORTING (i) Older YP were less willing to report SH. (ii) Boys were more likely to report SH than girls (iii) YP's beliefs about other people's stigma towards SH was a stronger predictor of help-seeking intentions	LOW RISK
Roberts-Dobie & Donatelle, 2007 [54]	US	To examine the experience, knowledge and needs of school counsellors in relation to students’ self-injurious behaviours	Quantitative- Questionnaire	443 school counsellors	Membership list of the American School Counsellor Association (ASCA)	REPORTING (i) Important for everyone to be educated around SH due to different individuals being involved in discovery (ii) Counsellors reported that they were the most appropriate contact (75%) and the most likely (77%). (iii) Specific facilitators to reporting- training, school policy, education, community connections, tangible support, and cooperation. (iv) Need for staff to build knowledge and skills. (iv) Cooperation from different organisations	REPORTING (i) School counsellors did not self-report high levels of knowledge on SH. (ii) Counsellors felt they didn’t know outside therapists enough for referrals. (iii)Lack of Time, Money, and Staff- School counsellors were asked to do more tasks in less time today and counsellors reported that they would need more time or more support staff	LOW RISK
Roberts, 2013 [59]	US	To develop knowledge by investigating experiences and practices of school counsellors working with YP who SH	Qualitative- Interviews	6 men, 6 women, aged between 30–64 years with 3–35 years of experience	Public and private schools in a South Eastern US state	RECORDING (i) Enlisting help to make referrals was important	REPORTING (i) Feeling uncomfortable/ uncertain- Some staff reported feeling uncomfortable and that it wasn't part of their role. (ii) Untrained or unwilling	MEDIUM RISK
Tillman et al., 2018 [60]	US	To understand the lived experiences of middle school girls who have engaged in NSSI and who have received help	Qualitative- Interviews using IPA	16 girls- mean age 13.8 years, 8th grade	Middle schools across the US	REPORTING- (i) YP valued being listened to, supported, and shown love. (ii) YP wanted to feel comfortable and have open and honest conversations about confidentiality, including what would be shared with guardians. (iii) YP wanted their staff to try to understand the reasons why they are engaging in self-injurious behaviours without judgement	REPORTING- (i)YP felt unsupported and unable/ reluctant to share. (ii) YP were afraid to seek help from the fear that their struggles would be dismissed. (iii) YP felt uncomfortable disclosing SH and were angry about how professionals addressed SH- feeling dismissed, misunderstood, or as if they were overreacting (iv) YP found it difficult to be honest and direct (v) Money- A barrier to YP disclosing was lacking health insurance	LOW RISK

**Table 4:** Table of the factors affecting the reporting or recording of self-harm within an online setting

Study	Country	Aim	Type of Study	Participants	Setting	Facilitators to Reporting/Recording	Barriers to Reporting/Recording	Quality Assessment
Frost et al. 2015 [55]	Australia	To explore experiences of YP who self-injure regarding online services, to inform online service delivery	Quantitative Question-naire	457 YP with a history of SH and responded to preferences for future online help-seeking	Online and offline sources- posters, flyers, first-year psychology students, youth mental health-websites, word of mouth, and social media	REPORTING- (i) 48.9% YP wanted to use the internet for help, 13.8% found help to speak with friends and family through shared experience. (ii) 71.1% had a smartphone and wanted to access help. (iii) Key themes- Information, guidance, online culture, facilitation of help-seeking, access, and privacy	REPORTING- (i) 35.1% didn’t want to speak to people in person, wanted to find all info online	LOW RISK

**Table 5:** Table of the factors affecting the reporting or recording of self-harm within an online setting

Study	Country	Aim of Study	Type of Study	Participants	Setting	Facilitators to Reporting/Recording	Barriers to Recording/ Reporting	Quality Assessment
Knowles et al. 2012 [61]	England	To explore YOT staff attitudes towards screening for SH in young offenders and referring them to specialist services, to identify potential barriers	Qualitative- Semi-structured Interviews	8 members of staff working in a city based YOT	Youth Offending Team	REPORTING (i) Passive screening- Methods of screening that didn't rely on the willingness of staff to perform screening for SH would be beneficial. (ii) Individual and organisational level- Needs to have a co-ordinated effort to remove barriers to screening (iii) Specialised training improves perceived confidence and knowledge	REPORTING (i) Staff not feeling qualified/ not part of their role- YOT staff often didn't feel comfortable talking about SH and that they didn't have the knowledge. (ii) Difficulties in making referrals to MH services- YP perceived stigma, lack of engagement with mental health services by YOT staff (iii) Not knowing how to help lack of confidence (iv) Limited time to help	LOW RISK

### Main findings

As mentioned, the thematic synthesis of the 19 included studies was considered from two perspectives: reporting and recording of self-harm. The resulting themes have been presented in Table 6

**Table 6:** Key themes from the included papers

Healthcare Setting		
	Facilitators	Barriers
Reporting	Co-ordinated approach Communication Guided self-help Individual care Information sharing Language Listening Staff experience Staff knowledge Training	Appointment length Communication Confidentiality Environment Failure to record Fear Ineffective screening Mental health literacy Not individualised care Parents Self-harm presentation Staff behaviour Staff experience Staff knowledge Staff perceptions Stigma YP characteristics
Recording	Coordinated approach Discussing recording	Information recording Stigma
School Setting		
Reporting	Co-ordinated approach Communication Different reporting methods Staff characteristics Staff experience Strategies to cope	Capacity Communication Confidentiality Limited resources Staff characteristics Staff education Staff experience Staff knowledge Staff perceptions Stigma Training
Recording	Coordinated approach Staff characteristics	
Online Setting		
Reporting	Help-seeking Internet use Smartphone use	Access
Recording		YP characteristics
Criminal Justice Setting		
Reporting	Passive screening Coordinated approach	Capacity Referrals Staff confidence Staff experience Staff knowledge
Recording		

As in Tables 2, 3, 4, 5 and 6, the results are presented by facilitators and barriers to reporting and recording, under the four settings included in the existing literature. These are as anticipated: healthcare settings [23, 47–49, 56, 57, 62, 63], school settings [25, 50–54, 58–60], criminal justice settings [61] and an online setting [55].

### Results—Healthcare setting

Eight papers demonstrated findings around the reporting and recording of self-harm in the healthcare setting [23, 47–49, 56, 57, 62, 63]. Bailey et al., is an editorial focusing on the challenges for general practice around self-harm in young people [23]. Bellairs- Walsh et al. explored young people's views and experiences related to the identification, assessment and care of suicidal behaviour and self-harm in primary care settings with GPs [57]. Fisher and Foster, looked at developing an evidence-based care plan/ pathway for children and young people in paediatric inpatient settings presenting with self-harm or suicidal behaviour [63]. Hawton et al., compared the characteristics of young people who reported deliberate self-harm episodes and presented at a hospital with those not attending hospital [47]. Jennings and Evans, explored the young person self-harm management and prevention practices, following reports that multi-agency teams were not effectively operating [56]. Saini et al., used Delphi methodology to reach consensus between different stakeholders and researchers on research priorities in suicide and self-harm to develop regional self-harm and suicide prevention and reduction schemes [48]. Miettinen et al. used different sources, such as an online forum to recruit young people to provide essays describing their experiences of healthcare related to self-harm in adolescence [62].The final paper by Tørmoen et al., sought to examine the use of child and adolescent psychiatric services (CAPS) by young people with both suicide attempts and non-suicidal self-harm, and to assess the psychosocial variables that characterised the young people [49].

#### Reporting facilitators

Facilitators to reporting self-harm across healthcare settings included recognising self-harm behaviours, training and experience, positive communication, individualised care, and safe information sharing. GPs being able to recognise behaviour when presented and initiating conversations around a young person’s self-harm, rather than the onus being on the young person, was seen as advantageous [57, 63]. Staff having a good knowledge and understanding around self-harm was seen as a reporting facilitator [63]. Although it was cited as being important to have received adequate training around self-harm [48], it was deemed to be more useful for clinicians to learn from their experiences of working with young people and understanding why they sought help [48, 56].

It was seen as imperative for young people to feel listened to, with an open dialogue [57], where they were given the opportunity to talk about their self-harm in their own words [23]. GPs using inviting and warm language, whilst demonstrating active listening with attentive body-language and good eye-contact were seen as facilitators [57]. In addition, young people valued being treated as an individual with GPs listening to young people’s concerns, preferences and offering them support as an individual. [56]. This was enhanced by clinicians informing young people around the outcomes of sharing information, in order to ensure that they felt comfortable and safe [57].

#### Reporting barriers

Barriers to reporting self-harm across healthcare settings included confidentiality concerns, negative perceptions of young people, communication difficulties, stigma and practical issues. Several studies found young people were often worried about reporting their self-harm to healthcare staff due to confidentiality and concerns around whether their information would be shared [23, 57]. This was compounded by young people experiencing poor mental health literacy and feeling hopeless and like they were a burden [57].

Poor communication was identified as being a barrier to reporting self-harm. Young people viewed the language, used by healthcare staff, around risk as problematic, ‘negative’ and ‘intimidating' [49], which is in line with other existing literature [26, 33]. In addition, GPs were viewed negatively if they appeared impersonal or indifferent towards young people. The language used by GPs was important in ensuring a young person felt they could report their self-harm and if it was not pitched appropriately, it could lead to missed opportunities for intervention [57]. Young people, who were self-harming, commonly had complex lives, with wider, confounding factors in play such as eating disorders and substance use and these were often underestimated [49, 56].

A prevalent theme emerged around stigma and perceptions; young people were reported as worried about not being taken seriously if they reported self-harm [23] and being concerned about the negative stigma associated with being labelled as a ‘self-harmer’ [48]. This was confirmed by findings in Fisher and Foster that reported young people who self-harmed were often labelled as ‘disruptive’, ‘demanding’, ‘aggressive’, and ‘difficult to understand and communicate with’ when presenting at hospital [63]. There were also negative perceptions of young people who self-harmed from specifically a care setting, reported in Jennings and Evans [56]. In the study by Miettinen et al. young people reported being unsure and uncertain about reporting their self-harm [62]. Young people felt anxious about their self-harm not being taken seriously and that they would ‘burden their loved ones’ [62]. Parents were also seen as a barrier for young people reporting self-harm. Often parents were unsupportive and reluctant about a young person reporting self-harm due to the negative connotations associated with a young person accessing psychiatric treatment [62]. Practical barriers to reporting self-harm included young people having to wait long periods of time for face-to-face appointments and the threshold to seek help was high as young people often required many GP visits and multiple referrals to access treatment [62].

Practical barriers to reporting self-harm across healthcare settings included too short appointments [23], ineffective screening tools that were not fit for purpose [23], hospitals as inappropriate settings for disclosing self-harm (chaotic, noisy etc.) [63] and non-individualised approaches hindering disclosure [56]. Within the hospital setting, nurses from the study by Fisher and Foster, expressed the desire for additional training to build knowledge as they reported feeling fearful of exacerbating young people’s problems [63].

#### Recording facilitators

There were fewer facilitators and barriers to recording self-harm in a healthcare setting extracted from the included papers. Facilitators were around being open to discussing what is recorded and services working together. Bailey et al. reported the importance of being able to discuss what was recorded in regards to a young person’s self-harm on their health records [23]. Talking to the young person about what was being recorded and for the staff recording it to be fully trained, was found to improve the consistency of recording [23]. In addition, Saini et al. highlighted the importance of different settings such as those within primary care GP and hospitals, schools and community services being able to communicate and work together when recording self-harm [48].

#### Recording barriers

The barriers to recording self-harm in a healthcare setting were mainly around stigma and the information being recorded. Often young people were found to be wary about the information around their self-harm being recorded and what information was shared and with whom due to the connotations of blame and associated stigma [23, 57].

The young people, involved in the study by Miettinen et al., reported that their self-harm was often ignored or professionals being unable to appropriately record self-harm and start the referral process [62]. In the study young people reported that their visible self-harm injuries were ignored and were not being recorded, despite them being asked about what they were [62]. This was compounded by staff not knowing what to record in relation to a young person’s self-harm and then not reacting after having seen visible injuries [62].

### Results—School setting

The setting where the most literature was found, with regards to the reporting and recording of self-harm in young people, was the school setting, where nine papers were included in the survey [25, 50–54, 58–60]. Most papers were around the willingness of young people to talk to school staff about their self-harm or how school staff reported or recorded self-harm and their attitudes around it. Berger et al. 2014, looked to validate a measure of attitudes towards Non-suicidal self-injury (NSSI) and examine the knowledge, attitudes, and confidence of school staff towards NSSI [50]. Dowling and Doyle, explored post-primary school guidance counsellors’ and teachers’ experiences of, and responses to, self-harm among students [58]. Evans et al. looked to ascertain whether young people who deliberately harmed themselves or had thoughts of self-harm differed from other young people in terms of help-seeking, communication and coping strategies [25]. Evans et al. sought to understand the school context, including existing provision, barriers to implementation, and acceptability of different approaches [51]. Heath et al. examined young people’s reports of willingness to access school-based support for NSSI [52]. Nearchou et al., determined the predictors of help-seeking intentions for symptoms of depression/ anxiety and self-harm in young people [53]. Roberts- Dobie and Donatelle, sought to examine the experience, knowledge and needs of school counsellors in relation to students' self-injurious behaviours [54]. Roberts et al. aimed to develop existing knowledge by investigating professional experiences and practices of school counsellors who work with young people who self-harm [59]. Finally Tillman et al., sought to understand the lived experiences of middle school girls who have engaged in NSSI and who have received professional help [60].

#### Reporting facilitators

Facilitators to reporting self-harm in young people, within the school setting, were staff being educated and knowledgeable, being able to make a young person feel comfortable, exploring different ways of disclosure and ensuring that staff well-being was also considered.

Multiple studies reported the importance of school staff being educated and knowledgeable around self-harm [51, 54, 58, 59]. More specifically, it was found to be advantageous to ensure the full spectrum of individuals, around a young person who is self-harming, to be educated, from teachers, counsellors, school nurses and other young people, parents etc. as they could all potentially be involved in reporting [54]. This also links with the finding around ensuring a co-ordinated approach was adopted, as joined up working helped to maintain consistent co-operation from different professionals [54, 59].

There was a plethora of results around who was the most appropriate member of staff for young people to report their self-harm to [25, 54, 58]. Roberts- Dobie reported that counsellors deemed themselves to be the most appropriate contact [54]. However, different members of staff being able to identify self-harm, in order to initiate conversations with young people, was seen as a facilitator to reporting. This included staff noticing subtle behaviour changes in a young person or being told about their self-harm by another young person, their friends or family Dowling et al. also reported the disclosure of self-harm could be via different subject teachers, with examples including English teachers identifying a young person’s self-harm via an emotive essay, or a Physical Education teacher noticing a young person’s refusal to change or wearing bandages to hide their injuries [58], rather than a defined member of staff being responsible for self-harm. Similar to the results from the healthcare setting it was seen as a facilitator for school staff to be open, non-judgemental and helpful [50, 60] and to ensure young people felt listened to [60].

Finally Dowling et al. reported the importance of school staff to have a way to debrief after difficult conversations with young people around their self-harm [58]. Staff found conversations less traumatic if they did not know a young person well, as they found it easier to maintain distance [58]. Self-care strategies, such as leaning on families, colleagues and friends and activities outside of work, also facilitated the maintenance of staff wellbeing [59].

#### Reporting barriers

Common barriers emerging from the included papers, within the school setting, were staff having a lack of knowledge around self-harm, a lack of time, money and resources, young people feeling uncomfortable with disclosing self-harm.

School staff commonly exhibited a lack of knowledge and confidence to help young people [50, 54, 58] and training was deemed to not be adequate [51, 58]. Dowling and Doyle reported that often school staff found self-harm difficult to identify, due to its hidden nature [58]. This was supported by Berger et al., which reported there was a need for helping young people to report their own or their peers’ self-harm [50]. There were role conflicts reported within some school staff, as some expressed being unwilling to participate in self-harm training as it made them feel uncomfortable and that it was not part of their role [59].

School staff were reported as feeling ‘panicked’ by self-harm reporting, but this reduced with experience [58]. Staff continuously faced concerns with larger class sizes and fewer yet busier teachers [58] with limited time and resources [51] and increasingly busy counsellors with heavier workloads [54]. This had a knock-on effect, resulting in less time for school staff to report self-harm.

Young people also felt reluctant and less comfortable reporting their self-harm to school staff [25, 60] and struggled with opening up and being honest, due to feeling like their concerns would be dismissed [60]. The reporting of self-harm was seen to be affected by a young person’s beliefs about other people's stigma towards self-harm and appeared to be a stronger predictor of help-seeking intentions than their own stigma beliefs [53]. This was exacerbated by school staff describing self-harm as ‘difficult’, ‘horrible’, and ‘disturbing’ in the study by Dowling and Doyle and the school staff were reported as being frustrated and less tolerant of young people perceived to be advantaged, with some staff considering self-harm behaviour to be ‘attention seeking’.

Age and gender differences were observed in regard to reporting. Heath et al. and Nearchou et al. which found younger school students, such as middle school students were more willing to report their self-harm and access school-based support than high school students [52, 54]. Nearchou et al. also found boys were more likely to report self-harm than girls [53]. Finally, money was cited as a barrier to disclosing or reporting self-harm, especially in countries such as the United States, as a young person was cited as being reluctant to report their self-harm due to not having health-insurance [60].

#### Recording facilitators

Again, there were fewer results focusing on the facilitators and barriers to recording self-harm. The facilitators that were identified were around age and co-ordinated help. Berger et al. reported that younger school staff were more knowledgeable and had higher self-perceived knowledge of NSSI than older colleagues [50]. This facilitated recording as they felt more comfortable doing so. Roberts et al. reported the importance of help recording a young person’s self-harm. Enlisting help to make referrals was found to be important and meant the process was more effective [59].

#### Recording barriers

The barriers to recording were around the length of professional experience and sex. Berger et al. reported the length of professional experience was negatively related to ability to identify NSSI, suggesting senior staff had poorer knowledge [50]. Staff who were more experienced with young people and NSSI were more confident and had higher self-perceived knowledge, understanding and more positive attitudes towards NSSI [50]. Therefore, suggesting a lack of experience responding to self-harm or an increased length of professional experience, were barriers to recording self-harm. The study also reported that females posed a greater confidence and knowledge of NSSI in comparison to males [50].

### Results—Criminal justice setting

Knowles et al. [61] looked at the staff attitudes, within a Youth Offending Team (YOT), around screening for self-harm in young offenders to identify potential barriers when referring young people to specialist services.

#### Reporting facilitators

Within the study by Knowles et al., there was only a focus around the facilitators and barriers to reporting self-harm by the YOT staff [61]. A facilitator was having the option to access passive screening. Having a self-harm screening process, that did not rely on the willingness of staff to perform screening, was seen as beneficial to reporting self-harm [61]. On an organisational level and similar to other settings, it was seen as important to have a co-ordinated effort from the individual to an organisational level to remove the barriers to screening [61].

#### Reporting barriers

Barriers to reporting self-harm included staff not feeling qualified or that it was not part of their role, time and difficulties making referrals [61]. Staff within the YOT often did not feel comfortable talking about self-harm with young people [61]. This was put down to staff feeling like they did not have the knowledge or experience with self-harm, not knowing how to help and it not feeling a part of their role. In addition some staff were not keen to engage with mental health services or they lacked the capacity or time to be able to do so [61].

### Results—Online setting

Frost et al. set out to investigate the perspectives of young people who self-injure regarding online services, with the aim of informing online service delivery, using a questionnaire [55]. It concentrates on young people who sought help for self-harm online, in order to determine their help-seeking preferences.

#### Reporting facilitators

Frost et al. found that using the internet was a facilitator to young people reporting self-harm [55]. Young people were reported as preferring to use the internet for help, over reporting their self-harm to someone in person [55]. This was amplified by their use of smartphones, and as the vast majority of young people included in the survey had a smartphone, they expressed wanting to access help using their phone [55]. Another key facilitator to reporting self-harm, and as reported in other settings was privacy [55]. Young people felt online sources were more private and would allow them to be freer to share their experiences.

## Discussion

Addressing the aim and the objectives of this systematic review; the findings will be able to support ongoing research and inform qualitative work with both young people who have self-harmed, their parents and the relevant practitioners. Across the different settings there were key themes that emerged around the reporting and recording of self-harm in young people, and these could be unpacked to identify both facilitators and barriers.

The theme of negative perceptions and stigma, associated with young people reporting their self-harm continued to be prevalent across the included papers. This is not a novel observation of this review, but a finding that has appeared consistently in the literature [64–66] and reviews of the evidence [67]. The negative perceptions associated with self-harm were seen as a significant barrier to both the reporting and recording self-harm as young people often felt anxious about what was being recorded and therefore felt uncomfortable reporting their self-harm [48]. It was seen as advantageous for staff to use positive communication techniques and warm, inviting language to facilitate reporting [57]. By ensuring honest and open conversations take place around self-harm and encouraging practitioners to raise topics, without young people needing to themselves, it would likely contribute to increased conversations and referral to treatment of self-harm [57]. Initiatives and campaigns, examples including ‘Self- injury awareness month’ annually in March [68], and Mind’s ‘Time to Talk’ [69] around mental health, can also be seen as tools for encouraging conversations around self-harm. Widespread coverage around self-harm can contribute to addressing the stigma and taboo associated with it and can ensure that standard and consistent messaging around self-harm is cultivated.

Another key theme was around the training, education and knowledge of different providers. Although this varied across the different settings and level of experience, it was evident that more still needs to be done to ensure all staff working with young people have the right tools to support them with reporting or recording self-harm [51, 54, 58, 59]. There appeared to be less focus around recording self-harm in the included studies, with most of the findings around recording being barriers, such as staff being unable or unwilling to record self-harm. Therefore, this suggests that individuals who work with young people who may be self-harming, should receive more comprehensive guidance and support around how to effectively record self-harm to ensure young people can be referred to the appropriate support services and that a standard approach to recording and referring is maintained. This could include system wide use of passive screening techniques, such as techniques that do not rely on the willingness of staff to perform screening for self-harm to prompt reporting, as referenced with the criminal justice setting [61].

Specifically within the criminal justice setting there appeared to be a conflicted role identity about it ‘not being part of the YOT workers role’ [61]. The focus should be on adopting a person-centred approach and moving away from the need to stick to defined roles when working with young people. This is especially fundamental when considering young people who self-harm, as often they can have stressful lifestyles, with a myriad of challenges, that affect all aspects of their life, including their education, relationships and behaviour [23]. Therefore, it would likely be beneficial for the full spectrum of staff and practitioners, working with young people, to be able to engage in conversations around self-harm [49, 56]. Future practice should also be centred upon organisations working together and communicating as this was a facilitator for both reporting and recording self-harm [48].

Language use was a key finding, and there was evidence around how important it was in ensuring that the language used around self-harm was appropriate, and how self-harm was talked about in conversations with young people [57]. Interestingly, there was no findings identified around the use of gender specific language and gender identity within the included studies. There was no exploration around how using more gender inclusive language, such as gender-neutral language, may facilitate conversations with young people, even though LGBTQ + young people have higher rates of self-harm and suicide than their cisgender, heterosexual peers [70]. Therefore, this indicates another valuable avenue for future study.

Practical barriers included the lack of time, money and resources [63] which remain problematic in the majority of current service provision. However, the internet and online services offered a way for young people to share information about their self-harm in a more private, controlled way with less input from professionals [55]. Therefore, future work should continue to tap into online support and ways to increase online provision further as smartphones and online technology continue to be a way of effectively communicating with young people, especially in a post COVID-19 pandemic world.

## Conclusion

From the systematic review of the current evidence, it was apparent that there is still progress to be made to improve the reporting and recording of self-harm in young people, across the different settings. Future work should concentrate on developing and implementing the facilitators including positive communication, joined up working approaches and exploring novel ways of reporting/ recording which engage young people, whilst aiming to ameliorate the barriers, such as the poor staff knowledge, stigma of self-harm and reducing concerns around information sharing. The findings of this review will also be able to support two ongoing research projects; (i) ‘Your Voice Heard’ where results from this study will be used to shape and inform the qualitative work with both young people who have self-harmed, their parents and the relevant practitioners, with the aim to provide recommendations for future practice, and (ii) the ADPH self-harm project, which is exploring case studies around young people who self-harm and giving a voice to school staff around current self-harm processes and procedures. For more information on either research project, please contact the corresponding author.

## Data Availability

All data analysed during this review are included in this published article.

## Abbreviations

ADPH: Association of Directors of Public Health
APPGSS: All-Party Parliamentary Group on Suicide and Self-Harm Prevention
ASCA: American School Counselor Association
CAPS: Child and Adolescent Psychiatric Services
CASP: Critical Appraisal Skills Programme
CINAHL: Cumulative Index to Nursing and Allied Health Literature
DSH: Deliberate Self-Harm
GP: General Practice
IPA: Interpretative Phenomenological Analysis
MeSH: Medical Subject Headings
MH: Mental Health
NSSH: Non-Suicidal Self-Harm
NSSI: Non-Suicidal Self-Injury
PE: Physical Education
PRISMA: Preferred Reporting Items for Systematic Reviews and Meta Analysis
PHE: Public Health England
RCP: Royal College of Psychiatrists
SH: Self-Harm
SPIDER: Sample, Phenomenon of Interest, Design, Evaluation, Research Type
UK: United Kingdom
US: United States
YOT: Youth Offending Team
YP: Young People
